# Applying Vernix Caseosa for Accidental Foetal Lacerations during Caesarean Delivery: A Case Series

**DOI:** 10.34763/jmotherandchild.20232701.d-22-00053

**Published:** 2023-08-31

**Authors:** Binarwan Halim, Hilma Putri Lubis, Timothy Adiwinata

**Affiliations:** Department of Obstetrics and Gynecology, Faculty of Medicine, Universitas Sumatera Utara, Medan, Indonesia; Stella Maris Women's and Children Hospital, Medan, Indonesia

**Keywords:** Foetal laceration, vernix caseosa, caesarean section

## Abstract

The caesarean section is a frequently performed method of delivery. Although the caesarean section is a low-risk and safe surgery, there is an increase in maternal and infant morbidity and mortality due to caesarean delivery. One of the most common infant morbidities is foetal laceration. Caesarean delivery has a 1–2% risk of laceration to the foetus. Various methods have been proposed to deal with laceration wounds. Studies have been conducted on vernix caseosa, which can heal wounds on the skin. This case series report aims to demonstrate that vernix caseosa application is a wound healing method that is highly effective, costless, and of immediate availability.

## Introduction

The caesarean section, or C-section, is a common and widely practiced method of delivery. Over the last few decades, the prevalence of the caesarean section has increased in both developed and developing countries. In Indonesia, a 10% increase (from 6.5% to 16.4%) in caesarean delivery was observed between the years 2007 and 2017 [1]. Although the caesarean section is a low-risk and safe surgery, there is an increase in maternal and infant morbidity and mortality due to the procedure [2,3]. The increased risks for neonates include lacerations, brachial plexus injuries, fractures, respiratory disorders, digestive system flora disorders, and allergies. The risk of laceration of the foetus during caesarean delivery is 1–2% [4,5,6]. It is an accidental injury that is not infrequent [7]. Lacerations occur more often during emergency caesarean sections than elective ones, most frequently occurring in patients with lack of progress in labour, ruptured membranes, oligohydramnios, or a very thin lower uterine segment [8].

### Case 1

A 29-year-old woman, gravida 2, para 0, abortion 1, was admitted to our emergency department with a premature rupture of membranes (PROM) that had lasted for 6 hours. General physical examination and vital signs were within normal limits. On pelvic examination, the following details of the first stage of labour were found: uterus enlargement appropriate for the gestational age, strong uterine contractions, cervical dilatation of 2 cm, negative amniotic fluid, singleton alive foetus, cephalic presentation, and increased foetal heart rate. The cardiotocography (CTG) examination showed foetal distress. The ultrasound examination performed at the delivery room demonstrated a greatly reduced amniotic fluid volume (with the amniotic fluid index, or AFI, being only 2.5 cm). No abnormalities were found in the laboratory results. We decided to perform an emergency caesarean delivery due to foetal distress.

**Figure 1.**
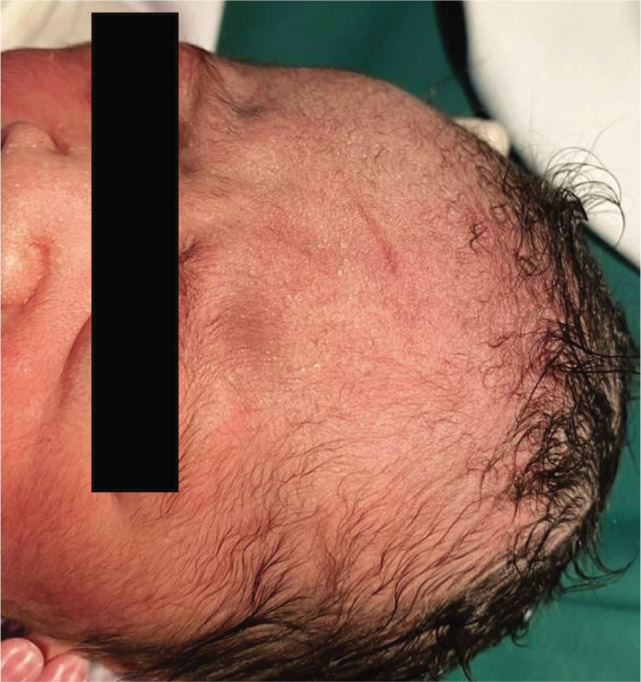
Laceration of 3 cm in length on the frontal region.

**Figure 2.**
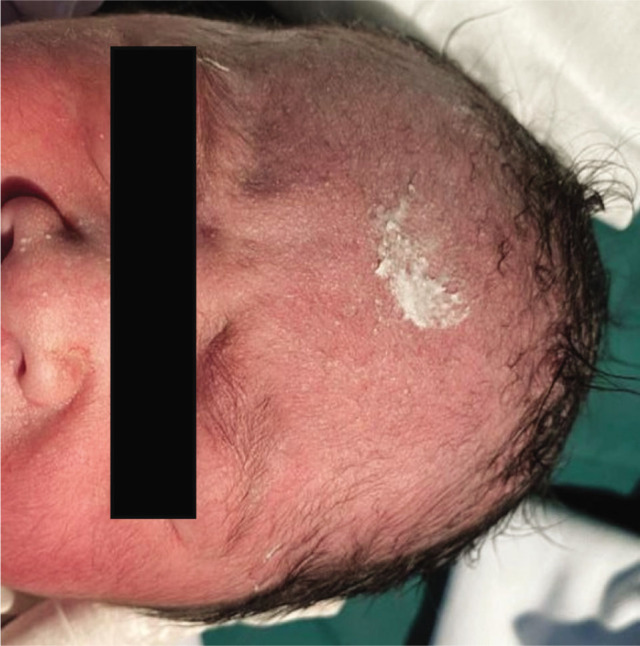
Wound closure with vernix caseosa.

**Figure 3.**
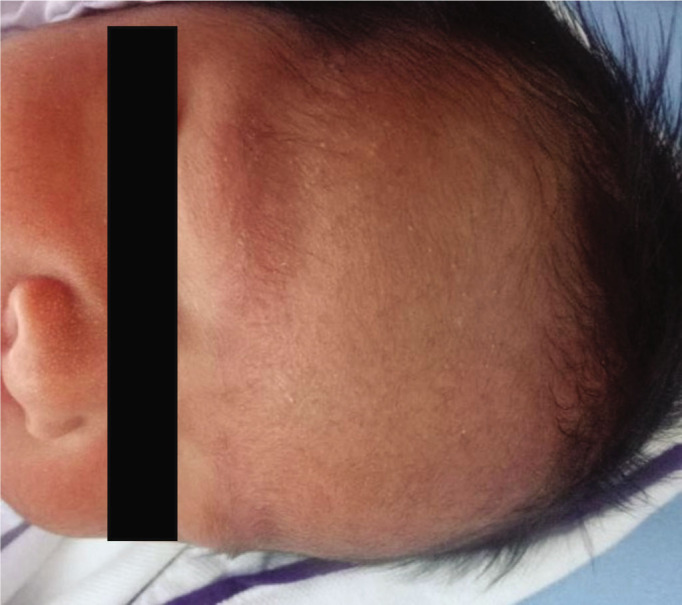
The outcome after treatment with vernix caseosa for 3 days.

When an incision in the lower uterine segment was made, the scalpel blade directly cut the frontal part of the baby's head, since the baby's head had passed the pelvic inlet at a greatly reduced amniotic fluid amount. As a result, the laceration in length of 3 cm on the baby's frontal region was noted (Figure 1). We decided to heal the laceration with vernix caseosa (VC) taken directly from the baby's body (Figure 2). The treatment with VC was maintained throughout the postpartum hospitalization period, which was 3 days. On Day 3, the VC was removed to evaluate the wound. The laceration turned out to heal without leaving a scar (Figure 3).

### Case 2

A 28-year-old primigravida presented at 40–41 weeks for her caesarean delivery with indications of breech presentation and postdate pregnancy. The general physical examination and vital signs were within normal limits. A pelvic physical examination revealed appropriate uterine size for gestational age. Cervical dilation was 0 cm with no regular contractions, amniotic fluid (+), singleton live breech presentation, and foetal heart rate within normal limits as confirmed by CTG. A recent ultrasound showed oligohydramnios. The laboratory tests were all normal. We decided to do an elective caesarean section.

**Figure 4.**
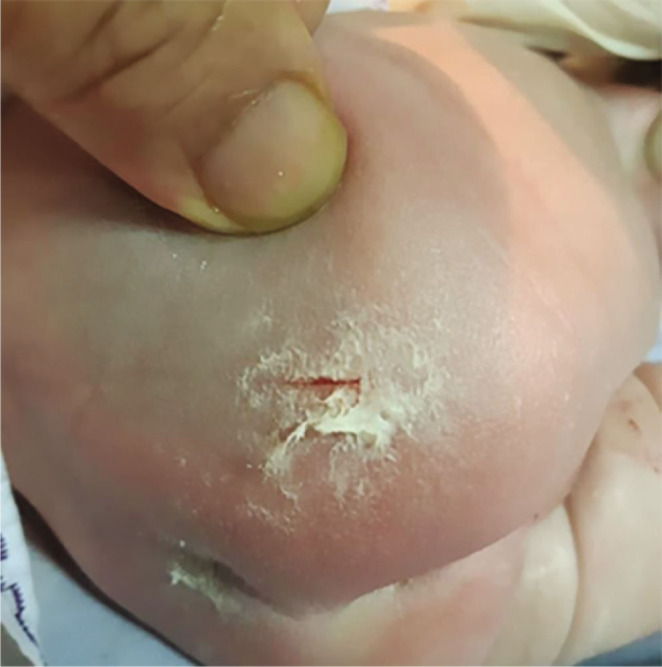
The right buttock with a laceration of 2 cm in length.

**Figure 5.**
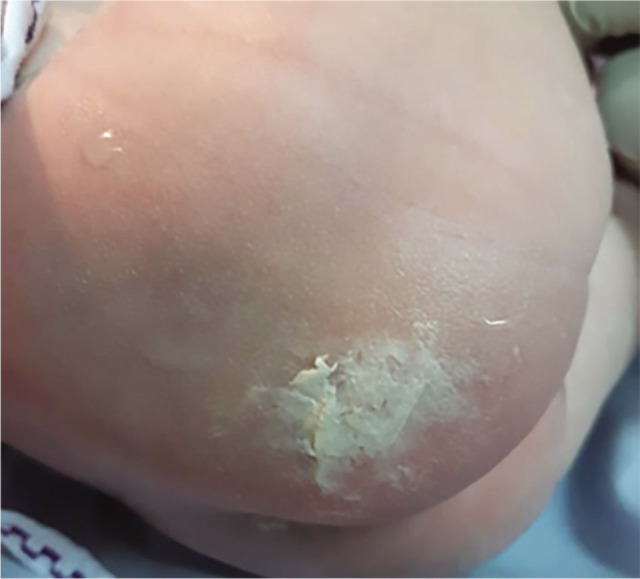
Wound closure with vernix caseosa.

**Figure 6.**
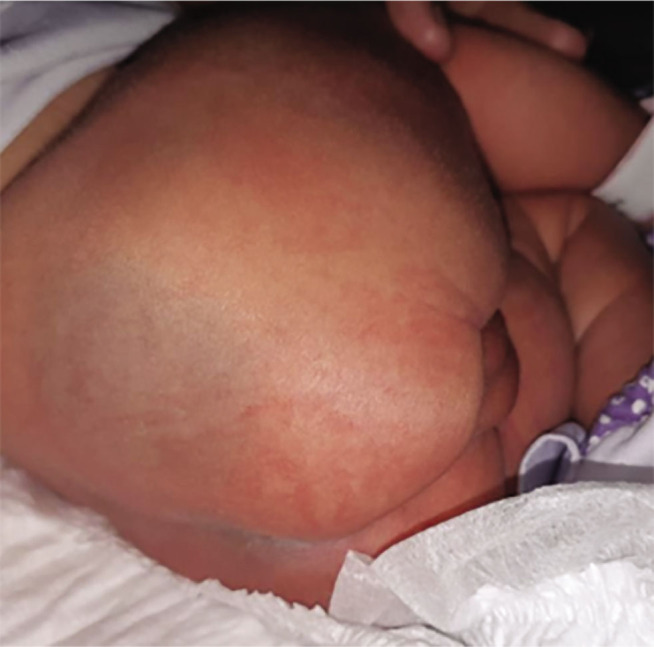
The result of a 3-day application of vernix caseosa.

The presenting part of the foetus (pelvis) was impinged behind the pubic symphysis. Using the pushing technique, an assistant used one hand to apply upward pressure inside the vagina to loosen the affected area and release it over the pubic symphysis. When an incision was made in the lower uterine segment, the scalpel struck the baby's right buttock directly (Figure 4). The incision wound was closed with VC that had been taken from the baby's body (Figure 5), then covered with a piece of plaster, and maintained as such throughout the mother's hospitalization. On Day 3, it was found that the incision wound on the baby's buttock had completely disappeared (Figure 6).

## Discussion

Caesarean sections are used for multiple maternal and foetal indications in order to reduce both maternal and infant morbidity and mortality. Complications associated with C-sections include increased risk of infection, blood transfusions, and prolonged hospital stay. A foetal laceration is an accidental injury that occurs during a caesarean delivery with a 1–2% risk [4]. The predisposing factors include: lack of progress in labour, PROM (as in our Case 1), oligohydramnios, thin lower uterine segment, history of caesarean delivery, foetal cephalic presentation, surgeon's inadequate skills, and gestational age above 33 weeks [8]. Lacerations in infants often occur in areas that can be aesthetically disturbing, such as the face. Although cosmetic defects are mainly found without functional impairment, these wounds may sometimes cause permanent scars on the baby's face [6]. Even minor lacerations limited to the skin layer can last up to 6 months after the injury. Prevention of accidental cuts to foetal skin during caesarean delivery must be widely implemented on a regular basis, with measures including effective blood evacuation at uterine incision and careful assessment of the thickness of the uterine incision site. Even with these prevention measures, the baby's body is still at risk for injury, especially in groups with the above risk factors. Therefore, various methods have been used to treat wounds that have occurred in babies during caesarean delivery, such as the use of skin adhesive tape, suturing, biologic glue, and creams [6,7,8]. Interestingly, VC is a white, thick, and viscous substance that has the same thickness as pasta or cottage cheese. It can be collected from virtually every newborn [9]. Moreover, VC consists of cells that are coated with lipids, and is biochemically a complex mixture of 80% water, 10% protein, and 10% lipid, including barrier lipids such as ceramides, free fatty acids, phospholipids, and cholesterol. It is produced by hair follicles during the last trimester of pregnancy, from head to toe and from back to front. Due to its lipid content, vernix is hydrophobic and protects the skin from excessive water exposure during the developmental phase of the stratum corneum layer of the skin, so that the baby's skin does not wrinkle while in the amniotic fluid [10]. Other functions of VC are to lubricate the skin so as to facilitate vaginal delivery, to prevent infection, to isolate the foetus from electrical conduction, to heal epidermal wounds, to regulate body temperature, and to support the development of the digestive tract [9,11,12]. There are studies on the wound-healing effects of VC, such as studies comparing VC with other creams in repairing the epidermis in controlled wounds, and studies on ceramides containing VC which can repair the skin layer [13,14]. In this report, VC was applied to the babies’ laceration wounds because it is a natural, costless material of immediate availability, and it can provide excellent wound-healing results. The mechanisms standing behind the wound healing by VC are stimulation of tissue metabolism, high glutamine content, and regulation of the trans-epidermal water gradient [15]. Glutamine is used as an energy source for cells to proliferate, including lymphocytes, macrophages, fibroblasts, and epithelial cells. [16] Compared to adult skin, neonatal skin has a fast wound-healing rate and a high potential for healing without scarring. In the first week of life, neonatal skin is highly reactive and adaptive due to differences in extracellular matrix composition, gene expression, cytokine response, and inflammatory response [17].

The use of VC taken directly from the baby's body surface to effectively heal its laceration from caesarean delivery has been applied at the Stella Maris Hospital, Medan, Indonesia, for approximately 10 years.

## Conclusions

Foetal laceration is one of the potential neonatal morbidities due to caesarean delivery. Such lacerations can be treated by applying VC taken directly from the baby's body surface. VC is a natural, costless material of immediate availability that provides excellent healing results for infant lacerations.
